# Quantification of left ventricular ejection fraction and cardiac output using a novel semi-automated echocardiographic method: a prospective observational study in coronary artery bypass patients

**DOI:** 10.1186/s12871-023-02025-z

**Published:** 2023-02-28

**Authors:** Thomas Komanek, Marco Rabis, Saed Omer, Jürgen Peters, Ulrich H. Frey

**Affiliations:** 1 Klinik für Anästhesiologie, operative Intensivmedizin, Schmerz- und Palliativmedizin, Marien Hospital Herne – Universitätsklinikum der Ruhr-Universität Bochum, Hölkeskampring 40, 44625 Herne, Germany; 2 Klinik für Anästhesiologie und Intensivmedizin, Universität Duisburg-Essen und Universitätsklinikum Essen, Essen, Germany

**Keywords:** AutoEF, Ejection fraction, Cardiac output, Pulmonary artery catheter, Echocardiography, Transthoracic echocardiography, Transoesophageal echocardiography, Haemodynamic monitoring

## Abstract

**Background:**

Echocardiographic quantification of ejection fraction (EF) by manual endocardial tracing requires training, is time-consuming and potentially user-dependent, whereas determination of cardiac output by pulmonary artery catheterization (PAC) is invasive and carries a risk of complications. Recently, a novel software for semi-automated EF and *CO* assessment (AutoEF) using transthoracic echocardiography (TTE) has been introduced. We hypothesized that AutoEF would provide EF values different from those obtained by the modified Simpson’s method in transoesophageal echocardiography (TOE) and that AutoEF *CO* measurements would not agree with those obtained via VTI_LVOT_ in TOE and by thermodilution using PAC.

**Methods:**

In 167 patients undergoing coronary artery bypass graft surgery (CABG), TTE cine loops of apical 4- and 2-chamber views were recorded after anaesthesia induction under steady-state conditions. Subsequently, TOE was performed following a standardized protocol, and *CO* was determined by thermodilution. EF and *CO* were assessed by TTE AutoEF as well as TOE, using the modified Simpson’s method, and Doppler measurements via velocity time integral in the LV outflow tract (VTI_LVOT_). We determined Pearson’s correlation coefficients *r* and carried out Bland–Altman analyses. The primary endpoints were differences in EF and *CO*. The secondary endpoints were differences in left ventricular volumes at end diastole (LVEDV) and end systole (LVESV).

**Results:**

AutoEF and the modified Simpson’s method in TOE showed moderate EF correlation (*r* = 0.38, *p* < 0.01) with a bias of -12.6% (95% limits of agreement (95%LOA): -36.6 – 11.3%). AutoEF *CO* correlated poorly both with VTI_LVOT_ in TOE (*r* = 0.19, *p* < 0.01) and thermodilution (*r* = 0.28, *p* < 0.01). The *CO* bias between AutoEF and VTI_LVOT_ was 1.33 l min^−1^ (95%LOA: -1.72 – 4.38 l min^−1^) and 1.39 l min^−1^ (95%LOA -1.34 – 4.12 l min^−1^) between AutoEF and thermodilution, respectively. AutoEF yielded both significantly lower EF (EF_AutoEF_: 42.0% (IQR 29.0 — 55.0%) vs. EF_TOE Simpson_: 55.2% (IQR 40.1 — 70.3%), *p* < 0.01) and *CO* values than the reference methods (*CO*_AutoEF biplane_: 2.30 l min^−1^ (IQR 1.30 - 3.30 l min^−1^) vs. *CO*_VTI LVOT_: 3.64 l min^−1^ (IQR 2.05 - 5.23 l min^−1^) and *CO*_PAC_: 3.90 l min^−1^ (IQR 2.30 - 5.50 l min^−1^), *p* < 0.01)).

**Conclusions:**

AutoEF correlated moderately with TOE EF determined by the modified Simpson’s method but poorly both with VTI_LVOT_ and thermodilution *CO*. A systematic bias was detected overestimating LV volumes and underestimating both EF and *CO* compared to the reference methods.

**Trial registration:**

German Register for Clinical Trials (DRKS-ID DRKS00010666, date of registration: 08/07/2016).

## Background

Assessment of the left ventricular (LV) ejection fraction (EF) plays an important role in perioperative risk stratification [1–4]. Various modalities of LVEF assessment have been developed, with transthoracic echocardiography (TTE) taking a leading role in everyday clinical practice due to its non-invasive nature and widespread availability. One of its common pitfalls, however, is the potential underestimation of cardiac dimensions. This phenomenon, known as foreshortening, occurs when the apex of the LV is not included in the ultrasound imaging plane due to an incorrect transducer positioning, causing the ventricular cavity to appear smaller [5, 6]. Furthermore, it is important that the endocardial border is clearly recognizable at end systole and end diastole. Reliable recognition of the endocardium, however, requires sufficient echocardiographic training and experience [7] and yet remains to some extent observer-dependent [8]. As a consequence, it would be desirable to introduce new, automated EF assessment methods so as to minimize the individual examiner influence.

Early semi-automated and automated methods were strongly dependent on 2D image quality and gain settings and hence found only limited adoption in clinical practice [9–11].

AutoEF (General Electric, Solingen, Germany) encompasses a speckle tracking based algorithm for semi-automated assessment of EF and other variables of LV function. Its principle rests on recognition of naturally occurring myocardial tissue patterns (‘speckles’) and analysis of their movement and deformation throughout the cardiac cycle. In contrast to the tissue Doppler, the velocity measurement is angle-independent and thus possible in any direction within the ultrasound imaging plane [12]. Suboptimal image quality, apical foreshortening or poor endocardial visualisation may limit both manual endocardial tracing and semi-automated methods as they occur during the image acquisition. However, it is unclear, whether speckle tracking based methods are able to compensate for some of these limitations by recognising image patterns invisible to the naked eye. Earlier studies showed a good correlation between AutoEF and the modified Simpson’s method in TTE [13, 14]. These studies were characterized by small and heterogeneous patient cohorts. Moreover, the time span between the echocardiographic examination and the chosen reference method was long or imprecisely specified and the intra-study variability was not taken into account. Finally, none of the studies examined the agreement of the AutoEF derived haemodynamic variables with the current clinical gold standard for haemodynamic monitoring, i.e., thermodilution *CO* by pulmonary artery catheter (PAC).

We, therefore, examined the agreement of AutoEF measurements with the two well established reference methods for EF and *CO* quantification in a larger group of patients. We hypothesized that AutoEF measurements would provide EF values different from those obtained by the modified Simpson’s method in TOE. We further hypothesized that AutoEF *CO* measurements would not agree with those obtained via VTI_LVOT_ in TOE and by thermodilution.

### Endpoints

The primary endpoints were the differences in EF and *CO* depending on their method of measurement.

The secondary endpoints were the differences in the left ventricular volumes at end diastole (LVEDV) and end systole (LVESV).

## Methods

### Study design

This prospective, partially blinded, non-interventional observational study was carried out between February 2016 and September 2017 in the Department of Anaesthesiology and Intensive Care Medicine at the University Hospital Essen, Germany.

### Patient recruitment

Three hundred and seventy-seven of 1,035 patients scheduled for elective cardiac surgery were screened for eligibility. Inclusion criteria were coronary artery disease (CAD) scheduled for elective CABG, sinus rhythm, absence of known valvular heart disease, age between 45 and 85 years, and ability to consent to study participation.

**Fig. 1:**
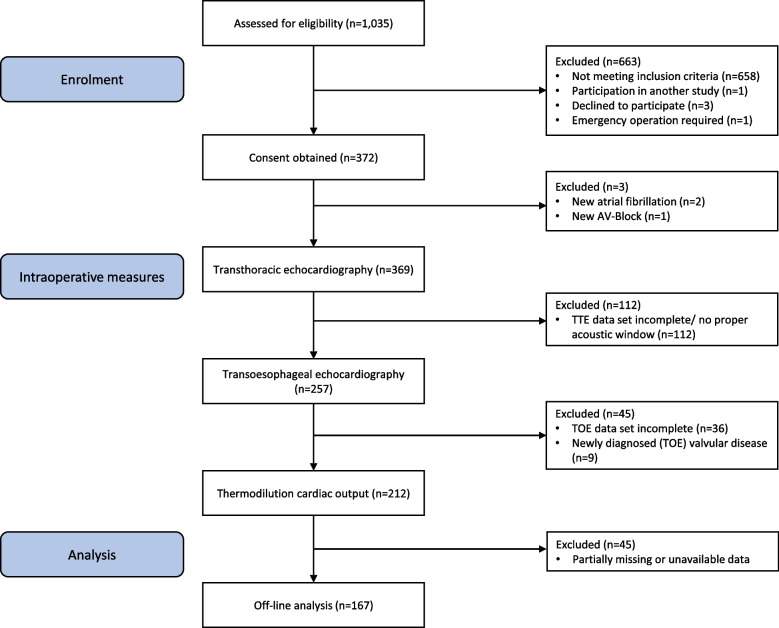
Patient selection and study flow diagram. TOE: transoesophageal echocardiography, TTE: transthoracic echocardiography

Exclusion criteria were refusal by the patient to participate, participation in another study, emergency surgery, atrial fibrillation, AV-block or valvular disease, incomplete TTE, TOE, or haemodynamic data sets and incomplete medical records. A study flow chart with the respective inclusion and exclusion criteria is depicted in Fig. 1.

Three patients refused to participate, and one patient was excluded because of participation in another study. Another patient had to be excluded since emergency surgery was required between the time of consent and the planned surgery. Additional patients were excluded due to newly diagnosed atrial fibrillation (*n* = 2), AV-block (*n* = 1), and valvular heart disease (*n* = 9) found only intraoperatively by TOE. Due to difficult scanning conditions, no adequate TTE and TOE images were obtained in 112 and 36 patients, respectively. Another 45 data sets were incomplete and thus a total of 167 patients with complete datasets were eventually analysed.

### Echocardiography and analysis

All echocardiographic examinations were performed with the Vivid S6 ultrasound system (General Electric, Solingen, Germany) using the M4S sector transducer for TTE and the 6Tc ultrasound probe for TOE. The data were postoperatively transferred to a central server and analysed offline using the Echo-PAC^©^ Clinical Workstation Software (General Electric, Solingen, Germany).

TTE was performed after anaesthesia induction but before surgery in the supine position and under stable haemodynamic conditions. To minimize effects of mechanical ventilation on cardiac preload and afterload, and to achieve the best possible image quality without artefacts, the examination was carried out during an end-expiratory breath-hold after prior ventilation with oxygen. While changing the transducer position from the apical four chamber (A4C) to the two chamber view (A2C), the patients were intermittently ventilated to maintain an expiratory CO_2_ tension around 40 mmHg.

Three ECG synchronized cine loops of each A4C and A2C view were stored. From these recordings, the cardiac cycle with the best image quality in a given imaging plane was selected for subsequent analysis, corresponding to recommendations of the American Society of Echocardiography and the European Association of Cardiovascular Imaging [15].

The AutoEF software processes 2D grayscale video sequences of transthoracic A4C and A2C views. First, three regions of interest in each imaging plane — the medial and lateral mitral valve annulus in A4C or the anterior and posterior mitral valve annulus in A2C, and the apex – need to be marked by the operator. This is followed by automatic detection of the endocardial border, which can be adjusted by the examiner, if necessary. The endocardial border is then traced throughout the whole cardiac cycle and the end systolic and end diastolic phases are recognized. After the loops in both imaging planes have been analysed and approved, automatically calculated biplane ejection fraction, cardiac output and other variables ​​are displayed.

The TOE examination followed a standardized shortened protocol based on the recommendations of the American Society of Echocardiography and the Society of Cardiovascular Anesthesiologists [16]. All TOEs were conducted within 15 min after completing the TTE. Recordings of the following imaging planes were obtained: midoesophageal four and two chamber view (ME 4C, ME 2C), midoesophageal aortic valve long axis view (ME AV LAX), transgastric long axis view (TG LAX) or deep transgastric long axis view (dTG LAX) with pulsed-wave Doppler (PWD) velocity measurement in the left ventricular outflow tract (LVOT). Three video sequences of each view were saved and the one with the best image quality was subjected to later analysis. When evaluating the Doppler measurements, the recording with the strongest signal was used.

According to the recommendations [17], LVOT diameter was determined in midsystole, 5 — 10 mm from the aortic valve and the average of triplicate measurements was calculated. Using pulsed-wave Doppler, the velocity of the LVOT blood flow was measured at approximately the same location in corresponding views and the average velocity time integral of three Doppler curves was used to estimate cardiac output, as suggested [18, 19].

Echocardiographic examinations were performed by attending anaesthetists (senior residents or consultants). Subsequent offline data analysis was carried out by the first author or one of the co-authors (MR, SO). To minimize bias, all study-relevant echocardiographic calculations were performed well after the end of the surgery. Hence, the observers were initially blind to AutoEF values when performing TOE and also unaware of VTI_LVOT_ *CO* when carrying out TDCO measurements. During the image analysis, however, the results of AutoEF measurements could not be hidden from the investigators before analysing TOE datasets for technical reasons.

### Thermodilution cardiac output

In compliance with local standard departmental operating procedures, a 5-lumen, 7.5 F, 110 cm pulmonary artery catheter (Edwards Lifesciences, Nyon, Switzerland) was introduced via the right jugular vein after anaesthesia induction and advanced until a pulmonary artery pressure tracing was obtained. Three boli of 10 ml ice cold 0.9% saline solution were injected during the TOE examination or immediately thereafter and mean cardiac output was calculated using the modified Steward-Hamilton equation [20]. The results of the *CO* measurements using AutoEF and TOE were unknown to the examiner.

### Statistics

Data are expressed as mean ± standard deviation (SD) in case of normal distribution of variables or median and interquartile range (IQR) in case of non-normality. To test for normal distribution, the Kolmogorov–Smirnov and the Shapiro–Wilk tests were used.

Normally distributed intergroup data were tested using the two-tailed unpaired Student t-test, otherwise the Wilcoxon test was used. Differences were considered statistically significant with an a priori two-tailed *p*-value of less than 0.05.

Values of variables derived by the respective methods were compared by calculating Pearson’s correlation coefficients *r* and fitting linear regression curves. Agreement of measurements was tested by the Bland–Altman analysis [21], calculating the bias with corresponding limits of agreement (95% SD of the mean difference). An EF bias of more than 10% was considered clinically relevant, as suggested by other authors [13, 22, 23]. As for *CO*, we accepted the 30% percentage error cut-off value proposed by Critchley and Critchley [24]. All statistical analyses were carried out with the SPSS software (Version 25, IBM, Armonk, USA).

## Results

After the initial patient exclusion based on predefined criteria, no further preselection took place, and 167 complete datasets were analysed. The patient cohort showed a clear prevalence of male sex (*n* = 145, 86.8%) and mean age was 69.6 years ± 9.8. Table 1 summarizes the demographic data.

**Table 1:** Demographics and preoperative clinical characteristics

Tab. 1	*n* = 167
Male, n (%)	145 (86.8)
Age (Years)	69.6 ± 9.80
Body height (cm)	174 ± 8.00
Body weight (kg)	85.1 ± 15.0
Body mass index (kg m^−2^)	28.1 ± 4.10
Systolic blood pressure (mmHg)	132 ± 17
Diastolic blood pressure (mmHg)	74.6 ± 11.7
Heart rate (min^−1^)	65.4 ± 12.5
**NYHA Classification, n (%)**	
NYHA 1	29 (17.4)
NYHA 2	70 (41.9)
NYHA 3	64 (38.3)
NYHA 4	4 (2.40)
**CCS Classification, n (%)**	
CCS 1	25 (15)
CCS 2	57 (34.1)
CCS 3	72 (43.1)
CCS 4	13 (7.80)
**Smoker, n (%)**	
Current	48 (28.7)
Past	59 (35.3)
**Diabetes mellitus, n (%)**	
Insulin-dependent	14 (8.40)
Non-insulin-dependent	37 (22.2)
Peripheral artery disease, n (%)	26 (15.6)
Chronic obstructive pulmonary disease, n (%)	29 (17.4)
Pre-existing pulmonary artery hypertension, n (%)	3 (1.8)
**Renal function, n (%)**	
Chronic kidney disease	48 (28.7)
Dialysis required	2 (1.20)
Myocardial infarction within past 90 days, n (%)	31 (18.6)
Immobility, n (%)	15 (9.00)
Cardiac output by thermodilution (l min^−1^)	3.98 ± 1.29
EuroScore 2 (%), median (IQR)	1,14 (1.00)

Data are presented as means ± standard deviation (SD) or absolute and relative counts. EuroScore2 is presented as median ± IQR

*NYHA* New York Heart Association, *CCS* Canadian Cardiovascular Society

### Ejection fraction

**Fig. 2:**
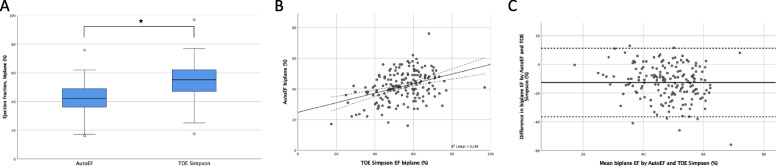
**A** Ejection fraction determined by transthoracic biplane AutoEF and TOE using the modified Simpson’s method. Box-and-whiskers plots show the first (bottom), second (inner line indicating median), and third (top) quartile. The whiskers represent the limits of 1.5 times the interquartile range. Outliers are depicted as circles below and above these limits. * *p* < 0.01. **B** Linear correlation of biplane EF determined by AutoEF and TOE using the modified Simpson’s method (*r* = 0.38, *p* < 0.01). Solid line represents the regression curve, dashed lines the mean confidence interval. **C** Bland–Altman plot for comparison of EF by biplane AutoEF and TOE using the modified Simpson’s method. Bias: -12.6%, 95%LOA: -36.5 — 11.3%. Solid line represents bias, dashed lines show the 95% limits of agreement (95%LOA). Data show a systematic EF underestimation by AutoEF. EF: ejection fraction, TOE: transoesophageal echocardiography

The medians of the transthoracic semiautomated biplane EF by AutoEF and the transoesophageal manual biplane EF using the modified Simpson’s method were 42.0% (IQR 13.0%) and 55.2% (IQR 15.1%; Fig. 2A), respectively (*p* < 0.01). The measurements showed a moderate correlation (*r* = 0.38, *p* < 0.01). However, the Bland–Altman analysis revealed a bias of -12.6% (95%LOA: -36.6 – 11.3%), suggesting an underestimation of EF by AutoEF compared to measurements using the modified Simpson’s method in TOE (Fig. 2B + C).

### Left ventricular volumetry

**Fig. 3:**
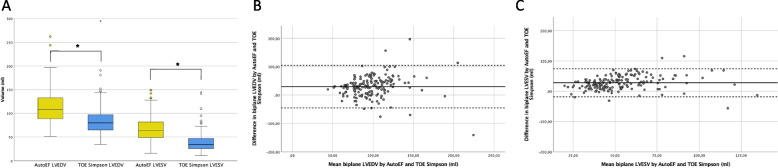
**A** Left ventricular end systolic (LVESV) and end diastolic volumes (LVEDV) as determined by biplane transthoracic AutoEF (yellow) and TOE using the modified Simpson’s method (blue). Box-and-whiskers plots show the first (bottom), second (inner line indicating median), and third (top) quartile. The whiskers represent the limits of 1.5 times the interquartile range. Outliers are depicted as circles below and above these limits, asterisks represent extreme outliers. * *p* < 0.01. **B** Bland–Altman plot for comparison of left ventricular end diastolic volumes determined by biplane transthoracic AutoEF and TOE using the modified Simpson’s method. Bias: 29.8 ml, 95%LOA: -45.3 — 105 ml. Solid line represents bias, dashed lines show the 95% limits of agreement (95%LOA). **C** Bland–Altman plot for comparison of left ventricular end systolic volumes as determined by biplane transthoracic AutoEF and TOE using the modified Simpson’s method. Bias: 28.1 ml, 95%LOA: -18.2 – 74,5 ml. Solid line represents bias, dashed lines show the 95% limits of agreement (95%LOA). EF: ejection fraction, TOE: transoesophageal echocardiography, LVEDV: left ventricular end diastolic volume, LVESV: left ventricular end systolic volume

Median biplane LV volumes calculated by AutoEF and using the modified Simpson’s method in TOE were 108 ml (IQR 45.0 ml) and 80.0 ml (IQR 32.0 ml) at end diastole and 64.0 ml (IQR 34.0 ml) and 34.0 ml (IQR 21.0 ml) at end systole, respectively (*p* < 0.01, Fig. 3A). The Bland–Altman analyses showed a bias of 29.8 ml (95%LOA: -45.3 – 105 ml, Fig. 3B) at end diastole and 28.1 ml (95%LOA: -18.2 – 74.5 ml, Fig. 3C) at end systole, respectively.

### Cardiac output

**Fig. 4:**
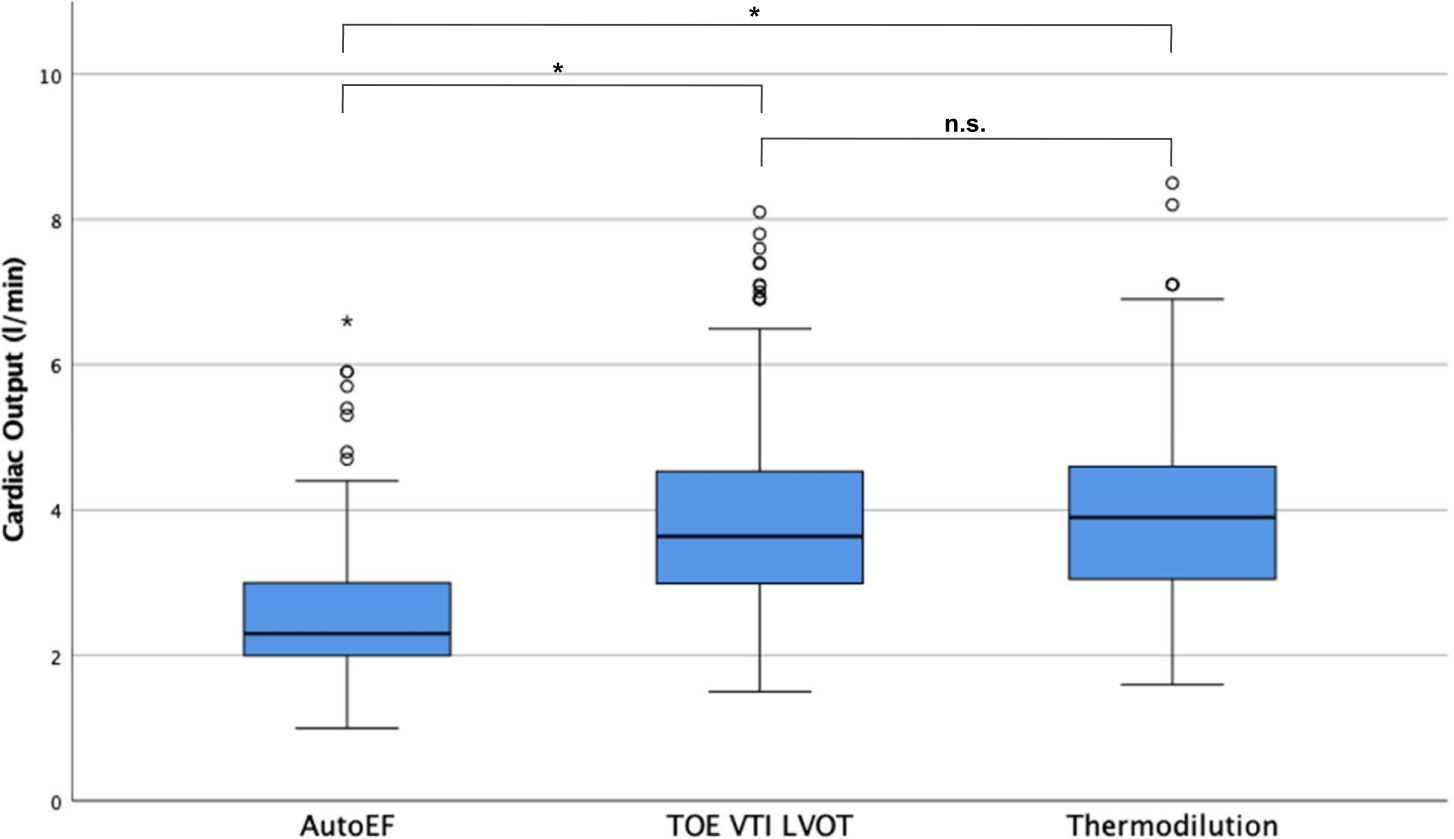
Cardiac output measurements by TTE AutoEF, TOE VTI_LVOT_, and PAC thermodilution. Boxes show the first (bottom), second (inner line indicating median) and third (top) quartile. The whiskers represent the limits of 1.5 times the interquartile range. Outliers are depicted as circles below and above these limits. The asterisk represents an extreme outlier. * *p* < 0.01. TOE: transoesophageal echocardiography, VTI: velocity time integral, LVOT: left ventricular outflow tract

Median *CO* measured by AutoEF, VTI_LVOT_ in TOE, and thermodilution were 2.3 l min^−1^ (IQR 1.0), 3.64 l min^−1^ (IQR 1.59), and 3.9 l min^−1^ (IQR 1.6), respectively. The AutoEF *CO* was significantly lower (*p* < 0.01) than those obtained with both other reference methods (Fig. 4). In contrast, *CO* derived from TOE VTI_LVOT_ and thermodilution did not differ (*p* = 0.328).

**Fig. 5:**
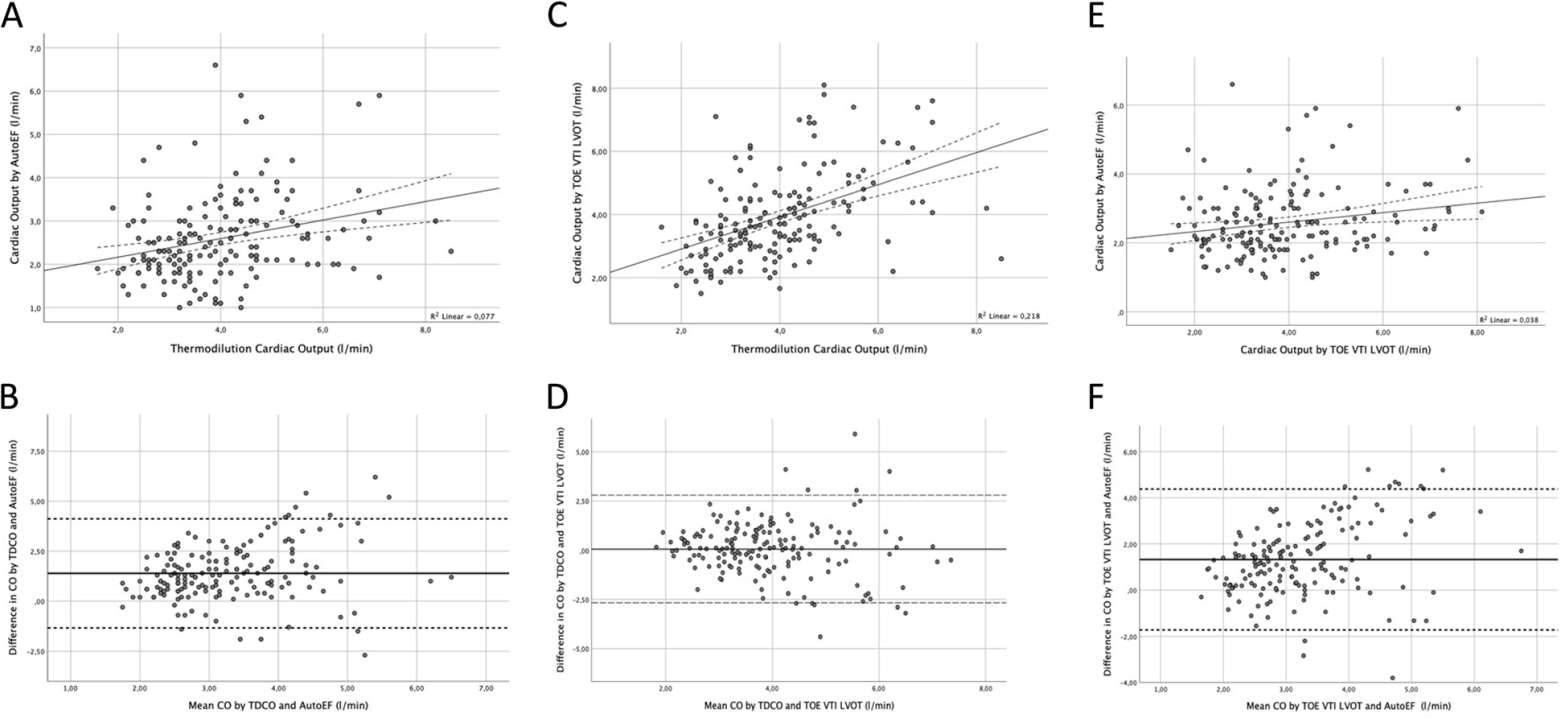
Linear correlations (**A**, **C**, **E**) and corresponding Bland–Altman plots (**B**, **D**, **F**) comparing cardiac output measurements by TTE AutoEF, TOE VTI_LVOT_, and thermodilution. For correlations, the solid lines represent the regression curves and dashed lines the mean confidence intervals. For Bland–Altman plots, the solid lines represent the bias and dashed lines show the 95% limits of agreement (95%LOA). CO: cardiac output, TOE: transoesophageal echocardiography, VTI: velocity time integral, LVOT: left ventricular outflow tract, TDCO: thermodilution cardiac output

A weak correlation was found between AutoEF *CO* and thermodilution *CO* (*r* = 0.28, *p* < 0.01, Fig. 5A). The Bland–Altman analysis revealed a bias as high as 1.39 l min^−1^ (95%LOA: -1.34 — 4.12, Fig. 5B).

AutoEF *CO* and the TOE Doppler *CO* also demonstrated a weak correlation (*r* = 0.19, *p* < 0.05, Fig. 5E) and the bias still was 1.33 l min^−1^ (95%LOA: -1.72 — 4.38, Fig. 5F).

In contrast, thermodilution *CO* and VTI_LVOT_ derived *CO* correlated stronger (*r* = 0.47, *p* < 0.01, Fig. 5C) and the bias was only 0.06 l min^−1^ (95%LOA: -2.67 — 2.80, Fig. 5D).

Thus, AutoEF correlated moderately with TOE EF as determined by the modified Simpson’s method and poorly both with VTI_LVOT_ and thermodilution *CO*. A systematic bias was detected, overestimating LV volumes and underestimating both EF and *CO* compared to the reference methods. On the other hand, TOE Doppler *CO* showed a good correlation with thermodilution *CO* and the bias between these latter methods was low and with much narrower limits of agreement.

## Discussion

Echocardiographic assessment of LV ejection fraction and *CO *play an important role in perioperative evaluation and can have far-reaching prognostic implications for patients with CAD [25]. Despite advances in 3D echocardiography and cardiac magnetic resonance imaging, 2D echocardiography is gaining popularity in routine clinical practice due to its widespread availability, point-of-care diagnostic capability, non-invasiveness, and relatively low acquisition costs [26–28].

EF quantification using the biplane modified Simpson’s method, as recommended by the echocardiographic societies, can be time-consuming and requires prior training as well as sufficient expertise of the operator [29]. As a result, a mere visual EF estimation is often used instead [30], which is, of course, fundamentally subjective. Nevertheless, studies have found a good correlation of this so-called ‘eyeballing’ with the disk summation method in experienced examiners [31]. Based on data analysis from previous studies, McGowan et al. reported an interobserver variability of 9 — 21% for the Simpson’s method and 8 — 17% for visual EF estimation, with a similar intraobserver variability of 6 — 13% for Simpson’s method and 11 — 13% for ‘eyeballing’. [8] Accordingly, there is room for improvement and new software tools for analysis of echocardiographic datasets could provide a possible solution, enhancing robustness and precision of quantitative EF evaluation. While first-generation algorithms for semi-automated LV evaluation proved unfeasible in clinical practise, novel speckle tracking based semi-automated and automated EF quantification previously showed good agreement and correlations with the Simpson’s disc summation method in transthoracic echocardiography [13, 14, 32]. In these studies, however, both methods were applied to the same set of echocardiographic data. In contrast, we compared a new, commercially available, semi-automated, speckle tracking-based software for transthoracic quantitative assessment of global LV EF with a different imaging approach, i.e., transoesophageal echocardiography, the latter known to provide a better acoustic window of the left ventricle. To our knowledge, no such analysis has been performed so far. In addition, we also compared AutoEF *CO* measurements with the TOE Doppler based *CO* using the LVOT velocity time integral and the time-tested gold standard – thermodilution via PAC.

The results of our study demonstrate poor agreement between AutoEF biplane semi-automated EF measurement and TOE biplane EF calculated by the modified Simpson’s method. The bias was greater than ten percent and with wide limits of agreement, indicating a clinically highly relevant underestimation of EF by AutoEF. In our opinion, such a discrepancy could possess significant implications for diagnostic and therapeutic decision-making, including indications for invasive preoperative cardiovascular procedures [33] or ICD-implantation [34], as well as volume therapy management or the administration of inotropic substances in ICU patients. Other authors proposed a possible benefit of supplemental AutoEF use for LV evaluation in less experienced examiners [14]. Considering our results, we cannot endorse such a proposal. AutoEF could provide inexperienced echocardiographers a false sense of security and, therefore, the results should always be interpreted with caution.

Our findings also correspond with prior observations made when another modality, i.e., cardiac magnetic resonance imaging, was utilised as a reference method [13, 35]. It seems that, due to differences in methodology and algorithms, LV measurements using different modalities, although each methodologically correct, may be partially influenced by the chosen method itself and, therefore, these modalities cannot be considered interchangeable [36].

For quantitative evaluation of echocardiographic data, acquired image quality is crucial regardless of the method used. In our study, manual correction of endocardial borders as detected by AutoEF and extrapolation due to suboptimal image quality were often necessary. Without this step, however, semi-automated image analysis would hardly have been possible at all.

According to current guidelines, transthoracic apical four and two chambre views should ideally be acquired in the left lateral position to maximise image quality and avoid foreshortening [37]. However, for several reasons, this recommendation is only achievable in cardiology echocardiography clinics but rarely in perioperative and ICU settings and, strictly speaking, there is only little formal evidence of improved TTE imaging quality in the left lateral compared with the supine position [38]. The anaesthetised patients in our study, therefore, remained supine during the examinations to reflect such perioperative conditions. This fact may have had a negative impact on the quality of the image recordings. On the other hand, if patient positioning were to have a significant impact on the EF calculation by AutoEF, this would impose a major limitation to its use in the above settings. Whether this is the case needs to be determined in future studies. Also, we did not use contrast enhancement even in patients with poor endocardial visualisation, as recommended by the American Society of Cardiology and the European Association of Cardiovascular Imaging in such cases [15].

In other comparative studies addressing quantitative assessment of the LV, the issue of endocardial delineation is often discussed [35, 39, 40]. The echocardiographic guidelines state that the LV cavity shall be traced along the endocardial border [15, 37]. However, due to trabecularization of the LV inner surface and subsequent image blurring, the boundary is more typically drawn through the trabecular tips when employing the modified Simpson’s method, possibly excluding a substantial amount of intraventricular volume from the calculation. Hence, a deeper, more aggressive delineation by AutoEF may have resulted in greater end systolic and end diastolic volumes, and lower EF values seen in our study.

Since the AutoEF *CO* calculation strongly depends on correct LV volumetry, it is not too surprising that its agreement with the respective reference methods was poor. The biases were as much as 1.39 l min^−1^, and thus also highly clinically significant in both cases, and the limits of agreement were wide. The lower *CO* derived from AutoEF compared to the other methods of measurement are also likely evoked by inaccuracies during TTE image acquisition and software related endocardial border detection.

On the other hand, the agreement between the two *CO* reference methods was good. The low bias and the limits of agreement were similar to those reported earlier [41] and confirm the quality of our TOE image material. Indeed, comparability of TOE Doppler *CO* using VTI_LVOT_ with TDCO has been the subject of several recent articles yielding mixed results [41–43] and both angular dependence of ultrasonic blood velocity measurements and the assumption of a constant circular rather than ellipsoid LV outflow tract were identified as important confounders for Doppler *CO *determination [44]. 2D echocardiography neglects the eccentricity of the LVOT, introducing a source of error and an underestimation of LVOT cross sectional area. Furthermore, 2D echocardiographic blood flow determination is imprecise due to incorrect assumption of a strictly axisymmetric and parabolic flow profile [45]. Thermodilution cardiac output measurements via pulmonary artery catheter still represent the most extensively validated clinical gold standard of haemodynamic monitoring and, therefore, a correct reference method in our study [46]. The fact that all patients received both TOE and PAC as per local SOP allowed a double validation of AutoEF CO against two well-recognized reference methods at no additional risk.

Although the utility of semi-automated quantitative echocardiography could not be confirmed in our study, we are still confident that this is a promising research area with significant clinical potential. With further development of 2D and 3D echocardiography and the implementation of artificial intelligence, advancements in this field can be expected in the coming years by improving imaging quality, enabling real-time user guidance and feedback during image acquisition, fully automated quantitative assessment, reduction of time needed to evaluate echocardiographic data sets, and increasing reproducibility and precision. Of course, apart from technological advancements, sufficient education, adequate training and certification play an essential role in echocardiography.

### Limitations

Our study has limitations. The echocardiographic examinations were performed by multiple observers with different levels of experience. All examiners had at least several years of expertise with perioperative TTE and TOE in a demanding setting of a high-volume, university-affiliated, tertiary care centre and all were supervised by board-certified examiners. According to our study protocol, all examinations using the three methods of assessment were supposed to take place within a short time span following anaesthesia induction and patient instrumentation in a stable state before surgical stimuli so as to minimize effects of haemodynamic alterations during measurements. Potential heart rate or blood pressure changes during each examination step were not analysed. However, under stable conditions prior to disinfection and incision such changes are likely minor and random. Further, whereas the TTE image acquisition was carried out during a short end-expiratory breath-hold, TOE images and thermodilution measurements were obtained regardless of the ventilation cycle. Right ventricular cardiac output, however, can vary across the ventilator cycle although such effects are negligible in normovolemic, intravenously prehydrated patients [47]. Three independent assessors were involved in quantitative image analysis. Although manual endocardial tracing inherently has a certain subjective potential, no intraobserver and interobserver variability were evaluated. Possibly, the omission of interobserver and/or interrater variability analyses is a limitation, as these may have provided insight into the degree of measurement heterogeneity and/or any potential effect of accessor experience. However, this was not the aim of the original study as we were more interested in the real-world daily practise. In addition, data interpretation was unbiased since the assessors were blind to other variables. Furthermore, the exact number of TTE datasets with suboptimal image quality, requiring manual adjustment of the detected endocardial border was not recorded. Only patients in sinus rhythm without valvular heart disease were included. Accordingly, no statement can be made about the value of AutoEF in patients with cardiac arrhythmias or valvular pathologies. Finally, although thermodilution *CO* represents the clinical gold standard, it is not an absolute *CO *reference gold standard. However, high-precision reference methods such as aortic root transit time difference ultrasonic flow probes are invasive, miss coronary blood flow, and are rarely used clinically [43].

## Conclusions

The validity of LV EF and *CO* assessment using AutoEF was not supported by our study. A systematic, clinically relevant bias was detected overestimating LV volumes and underestimating both EF and *CO* when compared with two established reference methods. Thus, AutoEF measurements and clinical decision making using this method should be considered with caution.

## Data Availability

The datasets used and/or analysed during the current study are available from the corresponding author on reasonable request.

## Abbreviations

2D: Two dimensional
3D: Three dimensional
A2C: Apical two chamber
A4C: Apical four chamber
AV: Aortic valve
CABG: Coronary artery bypass graft surgery
CAD: Coronary artery disease
CCS: Canadian Cardiovascular Society
CO: Cardiac output
dTG: Deep transgastric
ECG: Electrocardiogram
EF: Ejection fraction
ICU: Intensive care unit
IQR: Interquartile range
LAX: Long axis
LOA: Limits of agreement
LV: Left ventricle/left ventricular
LVEDV: Left ventricular end diastolic volume
LVESV: Left ventricular end systolic volume
LVOT: Left ventricular outflow tract
ME: Midoesophageal
NYHA: New York Heart Association
PAC: Pulmonary artery catheter
PWD: Pulsed wave Doppler
TD: Thermodilution
TG: Transgastric
TOE: Transoesophageal echocardiography
TTE: Transthoracic echocardiography
VTI: Velocity time integral
